# Is Surgery in the Elderly for Oesophageal Cancer Justifiable? Results from a Single Centre

**DOI:** 10.1155/2013/609252

**Published:** 2013-09-24

**Authors:** A. Mirza, S. Pritchard, I. Welch

**Affiliations:** ^1^Departments of Gastrointestinal Surgery and Histopathology, The University Hospital of South Manchester, SouthMoor Road, Wythenshawe, Manchester M23 9LT, UK; ^2^Department of General Surgery, University Hospital South Manchester, Southmoor Road, Manchester M23 9LT, UK

## Abstract

*Aims*. Advanced age is an identified risk factor for patients undergoing oncological surgical resection. The surgery for oesophageal cancer is associated with significant morbidity and mortality. Our aim was to study the operative management of elderly patients (≥70 years) at a single institute. *Methods*. The data was collected from 206 patients who have undergone operative resection of oesophageal cancer. The demographic, operative, histological, and postoperative follow-up of all patients were analysed. *Results*. A total of 46 patients of ≥70 years who had surgical resection for oesophageal cancer were identified. Patients ≥70 years had poor overall survival (*P* = 0.00). Also elderly patients with nodal involvement had poor survival (*P* = 0.04). Age at the time of surgery had no impact on the incidence of postoperative complication and inpatient mortality. Both the univariate and multivariate analyses showed age, nodal stage, and positive resection margins as independent prognostic factors for patients undergoing surgery for oesophageal cancer. *Conclusions*. Advanced age is associated with poor outcome following oesophageal resection. However, the optimisation of both preoperative and postoperative care can significantly improve outcomes. The decision of operative management should be individualised. Age should be considered as one of the factors in surgical resection of oesophageal cancer in the elderly patients.

## 1. Introduction

The oesophageal cancer is the eighth most common cancer worldwide, with 481,000 new cancers estimated in 2008 and the sixth most common cause of cancer death [1]. In the UK, annually there are approximately 7,800 new cases diagnosed and 7,000 deaths which result from oesophageal cancer [2]. It is associated with poor five-year survival rates of 10 to 20%. Over 80% of patients suffering from the disease are more than 60 years of age [2]. Thus, most patients who undergo treatment for oesophageal cancer fall into an older age group [3]. Because of longer life expectancy, self-awareness, availability of modern diagnostic modalities, advancement in treatment options, and modern surgical practice, more patients are being diagnosed and referred for surgical management. Treatment is based on initial staging of the oesophageal cancer. In the UK for both common histological subtypes of oesophageal cancer (adenocarcinoma and squamous cell carcinoma), if a patient is considered fit for resection, they will also be considered for perioperative chemotherapy (OEO2 [4], MAGIC [5]). In the UK a preoperative course of radiotherapy is not given routinely.

Advanced age is identified as an independent prognostic factor in patients undergoing surgery for cancer with increased rate of perioperative morbidity and mortality [6, 7]. The incidence of cancer in elderly patients is 10 folds higher in comparison to younger age group [8]. The risk to develop invasive cancer for older males (44%) is higher than that for females (38%) [8, 9]. Esophagectomy is a major surgical procedure and is associated with significant risk of postoperative complications. There are conflicting reports about the management of oesophageal cancer in the elderly population and also the effect of advanced age and performance status on the overall prognosis in the elderly patients undergoing curative resection for oesophageal cancer [9, 10]. The aim of this study was to evaluate the outcome for elderly patients undergoing potentially curative surgical resection for oesophageal cancer at a specialist gastrointestinal oncology unit. There is no cut-off to define elderly age group. In most of the published literature age of ≥70 years has been used to describe older patients [10, 11].

## 2. Patients and Methods

The data was collected prospectively for all patients who underwent curative surgical resection for oesophageal and gastrooesophageal junction tumours between 1996 and 2010 (*n* = 206) at the University Hospital of South Manchester. All patients had received standard preoperative workup which included clinical assessment physical examination, laboratory investigations, lung function tests, anaesthetic assessment and radiological imaging. The standard radiological investigation was a staging CT abdomen, pelvis, and chest. Nuclear imaging PET-FDG or PET-CT scan was performed when it was clinically indicated. Endoscopic ultrasonography was routinely performed for all patients from 2006. All patients were preoperatively assessed by a specialist anaesthetic team. The preoperative risk was assessed using the American Society of Anaesthesiologists (ASA) criteria. Patients diagnosed with gastrooesophageal junction tumours underwent staging laparoscopy to assess the spread of the disease.

All patients diagnosed with oesophageal cancer were discussed in a multidisciplinary cancer meeting. Patients who were deemed not fit for surgical resection were referred for radical chemoradiotherapy or palliative treatment.

Since 1996, our surgical department has participated in both OEO2 and MAGIC chemotherapy trials conducted by the MRC (UK) and the results were later published in 2002 and 2006, respectively. The neoadjuvant chemotherapy was administered to patients who meet the criteria. OEO2-based chemotherapy was administered for both squamous and adenocarcinoma of the upper and middle thirds of oesophagus [4]. Patients with lower third of oesophagus and gastro-oesophageal junction were considered for chemotherapy following MAGIC protocol [5]. 

## 3. Surgery and Postoperative Course

The type of surgical procedure was based on the site of tumour and surgeon's preference. In most cases, an Ivor-Lewis (IL) oesophagectomy (middle, lower thirds, and gastro-oesophageal junction) or left thoraco-abdominal [12] approach (middle and lower thirds, oesophagus and GOJ) was performed both incorporated a two-field lymphadenectomy. The feeding jejunostomies were sited to support nutrition in the early postoperative period. All patients postoperatively were transferred to high dependency unit and managed by a specialist team. Patients were regularly seen by a dietitian and hospital nutrition team. Patients were encouraged to mobilise early; regular chest physiotherapy to prevent lower respiratory tract infection and all invasive catheters and vascular access lines were removed as soon as possible. Both in-patient 30-day mortality and morbidity were recorded.

## 4. Follow-Up

All patients were closely monitored for their duration of the stay in the hospital. Following successful recovery, patients were discharged with out-patient follow-up at 15 days and 1, 3, 6, and 12 months. After the first year, all patients had 6-months follow-up. Patients who received neoadjuvant chemotherapy were administered adjuvant chemotherapy within 12 weeks according to MAGIC protocol. Patients with recurrent disease were assessed for consideration of palliative therapy. 

The demographic details of patients including age, gender, survival status, and follow-up data were collected. The dates of tumour recurrence and subsequent management were also recorded. The patient's disease-free survival was defined as the time from surgery to the time of recurrence or death without recurrence. Overall survival was defined as the date of surgery to the date of last follow-up or death. 

## 5. Histopathological Analysis

A minimum histological dataset was developed to collect the histopathological characteristics of oesophageal cancer following curative surgical resection. The data collected for each patient included the tumour site, TNM stage, and tumour differentiation. TNM 6 classification was used to assess the tumour stage. Both the proximal and distal resection margins were examined histologically to asses complete tumour resection. The involvement of circumferential resection margins (CRM) was also examined.

## 6. Statistical Analysis

Patients were divided into two groups based on age at the time of surgery (<70 years, ≥70 years). The demographic characteristics and histological findings were compared between the two groups using Chi-square (*χ* ^2^) and Fisher's exact tests. Relationships to prognosis were calculated using univariate and multivariate Cox-proportional hazard models. Survival curves were plotted using the Kaplan-Meier method and the log-rank test was used to determine the significance. A *P* value <0.05 was considered statistically significant. All data were analysed using SPSS version 16.0 (SPSS Inc., Chicago, IL, USA).

## 7. Results

Data was collected for 206 patients who underwent surgical resection for oesophageal carcinoma. Forty six (22%) patients were ≥70 years and 160 (78%) patients were <70 years at the time of surgery. Both dysphagia and weight loss were the most common presenting complaint for patients (Table 1).  Table 2 describes the general demographic characteristics of the patient population. 76% of all patients had tumour located at the distal oesophagus and gastrooesophageal junction. More patients aged <70 years were treated with neoadjuvant chemotherapy than patients aged ≥70 years. There was no difference in tumour recurrence among the two patient age groups.  Table 3 details the median age, overall survival, and time to recurrence of both groups. There was no difference in the tumour stage, nodal involvement, differentiation, status of circumferential resection, and involvement of proximal resection margins (Table 4). Importantly, there was no significant difference in the postoperative morbidity and mortality in the two age groups (Table 5). 

Age >70 years, nodal stage, and involvement of longitudinal resection margins were identified as independent prognostic factors in patients undergoing curative resection for oesophageal cancers (Table 6). 

**Figure 1:**
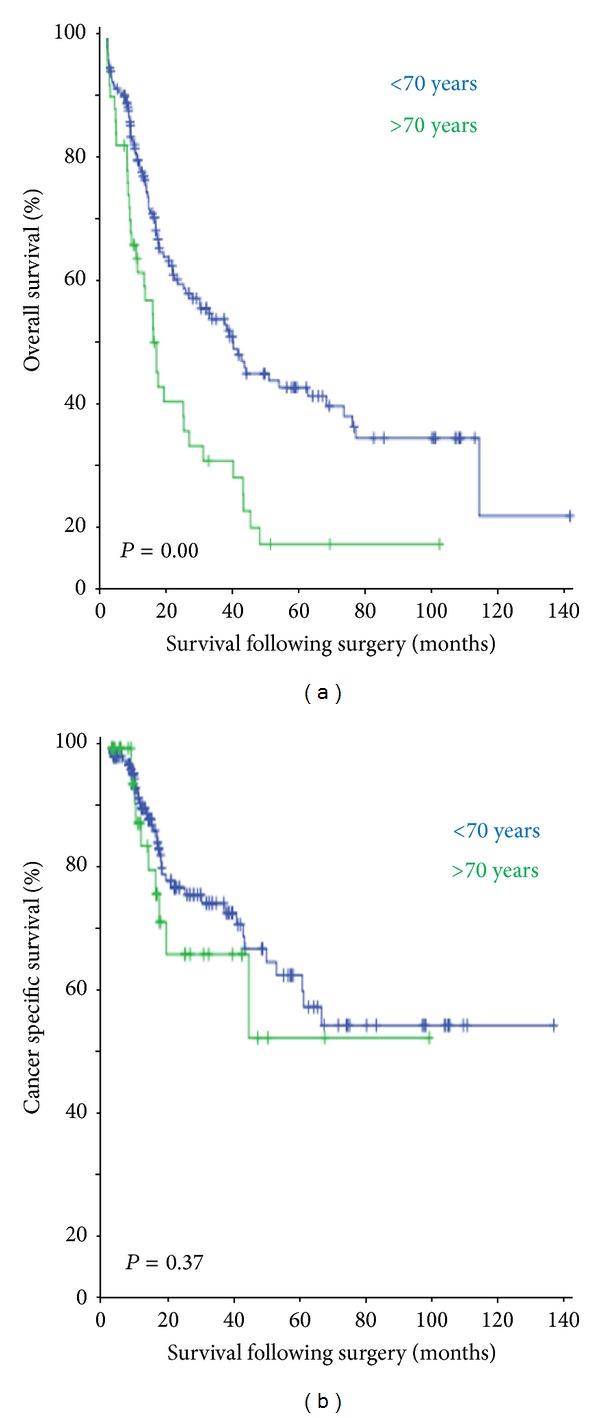
The overall survival (months) and cancer-specific survival (months) of the two groups undergoing oesophageal resection. The overall survival is significantly worse in patients of ≥70 years. There is no difference in the cancer-specific survival between the two groups.

**Figure 2:**
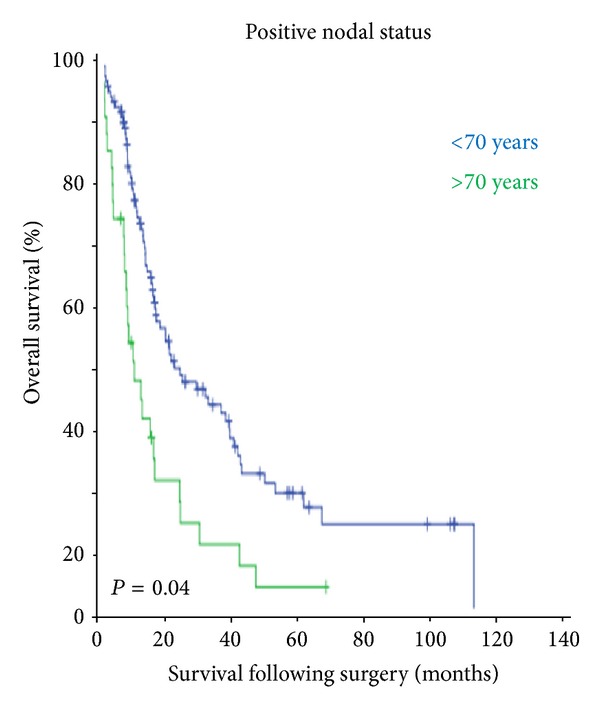
The nodal positive status is associated with significant poor survival in the elderly patients.

**Figure 3:**
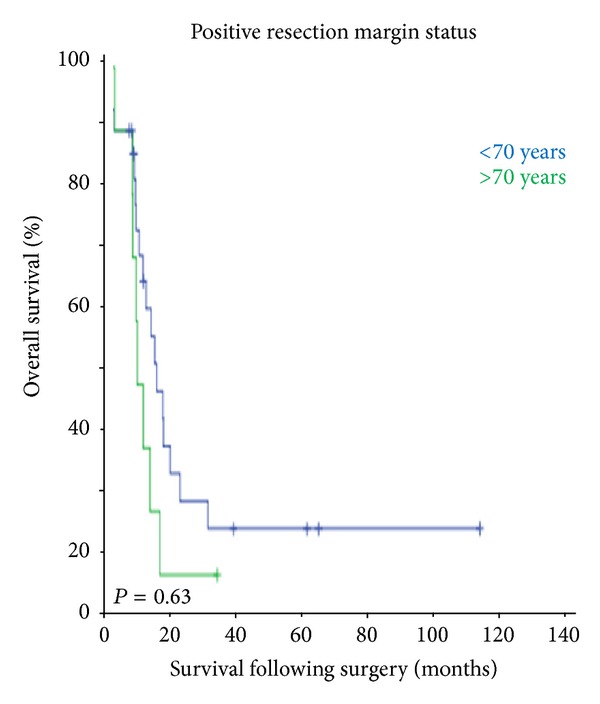
There is no difference in survival in patients with positive resection margin status. The tumour involvement of the resection margins is associated with tumour recurrence and poor survival.

Figure 1 shows the overall survival and the cancer-specific survival of the study population. Despite similar postoperative morbidity and mortality in the two groups, patients of ≥70 years had poor outcome following oesophageal resection (*P* = 0.00). Also elderly patients who had undergone surgery and histology examination showing positive lymph nodes had poor survival as compared to younger patients (*P* = 0.04) (Figure 2). There was no statistically significant survival difference between the two age groups if there was tumour involvement of the longitudinal resection margins (Figure 3). 

## 8. Discussion

Over recent decades, there has been a gradual increase in the population aged ≥70 years. As a result more patients in this age group are being referred to oncology surgical teams for consideration of surgery. This has become a major health concern for physicians dealing with elderly patients to treat or not to treat [10]. Oesophagectomy is a major surgical procedure associated with well-recognised morbidity and mortality. Recent advances in both preoperative and postoperative management, of elderly patients undergoing surgical procedures have improved outcome. There is no consensus on suitability of elderly patients to undergo oesophageal resection [12–16]. Both advanced age and reduced pulmonary reserve are predictors of poor outcome following oesophageal resection [17]. Several recent studies have concluded a favourable outcome in patients of ≥70 years undergoing oesophageal resection and have found comparable operative course, postoperative complications, and overall survival compared with younger patients (age <70 years) [10, 13, 18]. But other studies have questioned both the short- and long-term outcome for oesophageal resection in elderly patients [16, 17]. The overall survival (median = 10 months) has been poor in the elderly [19]. 

Aging is characterised by the decline in physiological reserve of the body. Elderly patients often have complicated medical background especially compounded by the presence of significant comorbidities [7]. The body's ability to mount physiological response against surgical trauma may be diminished. The immune system can be impaired and older patients may have a tendency to develop complications earlier. 

The optimal preoperative evaluation of patients by a specialist multidisciplinary team is a key factor which can improve surgical outcome. Pulmonary complications including atelectasis, lower respiratory tract infection, pulmonary oedema, pleural effusion, and adult respiratory distress syndrome are the most frequent causes of morbidity and mortality. Poor pulmonary function further leads to hypoxemia and hypercapnia [20]. These could be improved by early postoperative mobilisation, chest physiotherapy, appropriate analgesia and deep breathing exercises. Cardiovascular disease is more common in the elderly patients and can also be a significant cause of postoperative morbidity [21]. This could be improved by the peri-operative input from the cardiology and anaesthetic team input in the optimisation of cardiac function and risk assessment for cardiac events [22]. 

The main question that has remained unanswered in the literature is the choice between curative surgery and chemoradiotherapy in the elderly age group. There are no large-scale prospective trials or clinical studies which have answered this question. Only few studies have compared surgery versus nonoperative management in oesophageal cancer in the elderly patients [15, 23]. A surgical procedure that can be safely completed and is well established does not necessarily mean that it should be performed as the only option [15]. The decision to treat surgically is a balancing act between risks and benefits and most favourable outcome for the patients.

Chemoradiotherapy is an alternative option to surgery in elderly patients for the management of oesophageal cancer. A clinical response has been observed in up to 65% of patients and a 2-year survival rate of up to 40% [24, 25]. Only a few studies have explored the potential of chemo-radiotherapy in elderly population and have identified favourable outcome. The results following CRT were comparable to younger population [13, 26, 27]. 

Other factors have been identified in the elderly patients which influence treatment outcome including more than 10% of body weight loss, WHO performance score >1, poor nutritional status, comorbidities, and age of >75 year have been described as factors which may dictate against operative management in the elderly patients suffering from oesophageal cancer [13]. 

Nodal involvement had been identified as an independent prognostic factor in the surgical management of the oesophageal cancer. Patients with the nodal involvement have poor survival and increased incidence of tumour recurrence [28]. In our series of patients, nodal involvement was similar in the two groups and there was no significant difference in survival in both age groups if the tumour was node positive (*P* = 0.05). The involvement of >20% of the total lymph nodes is marked with poor prognosis. Similarly, patients who had involvement of the longitudinal resection margins independent of their age group had poor survival. The involvement of the resection margins is associated with tumour recurrence, anastomotic leakage, and poor survival. 

The data we have presented is limited by the fact that it includes only patients who have undergone oesophagectomy following strict selection criteria for consideration for surgery. It does not include both young and elderly patients who were not offered surgery because of their underlying co-morbidities or patients refusal to treatment. It also does not include or compare patients who chose not to have surgery and were treated with other modalities.

It is recommended that oesophageal resection should preferably be performed in a specialist regional centre to improve both morbidity and mortality [29, 30]. A recent study concluded that a minimum of 13 oesophagectomies each year should be performed in the respective hospital and the increased patient volume was related to better outcome [31].

The oesophagogastrectomy can be performed in the elderly patients with acceptable morbidity and mortality. It would be recommended that importance should be given to careful selection of patients suitable for surgical resection. Though advanced age is associated with poorer outcome, it should not be the deciding factor. A multidisciplinary approach combined with best clinical care can help to improve outcome in both younger and older patients. The decision should always be individualised and discussed with the patients, including their expected perioperative course and outcome. 

## Figures and Tables

**Table 1:** Presenting symptoms.

	<70 years (*n* = 160)	≥70 years (*n* = 46)
Dysphagia	122 (76%)	31 (67%)
Weight loss	38 (23%)	12 (26%)
Epigastric pain	12 (7%)	2 (4%)
Anaemia	8 (5%)	3 (6.5%)
Dyspepsia	16 (10%)	3 (6.5%)
Heart burn	5 (3%)	2 (4%)

**Table 2:** Patient characteristics.

		<70 years (*n* = 160)	≥70 years (*n* = 46)	*P* value
Gender	Male	137	37	0.53
	Female	23	9	
Tumour site	Oesophagus	42	7	0.85
	GOJ	118	39	
ASA grade	1	20	6	0.89
	2	96	27	
	3	42	13	
	4	2	0	
Type of Surgery	IL	85	16	0.02
	LTA	75	30	
Neoadjuvant chemotherapy	Yes	87	9	0.003
	No	73	37	
Tumour recurrence	Yes	68	21	0.83
	No	92	25	

GOJ: Gastrooesophageal junction.

**Table 3:** Age, overall survival, and time to recurrence.

	<70 years			≥70 years		
	Median (years)	Range (years)	95% CI	Median (years)	Range (years)	95% CI
Age	60.5	36 to 69	57 to 64 years	73.1	70 to 82	73 to 75
Overall survival	1.3	<1 month to 11.5 years	24 to 34 months	0.9	<1 month to 8.3 years	12 to 24
Time to recurrence	0.8	2 months to 6 years	13 to 18 months	1.0	2 months to 5 years	13 to 20

**Table 4:** Histopathological characteristics of the resected tumour specimens.

		<70 years (*n* = 160)	≥70 years (*n* = 46)	*P* value
Tumour type	ACC	152	45	0.68
	SCC	8	1	
Tumour differentiation	Well/moderate	89	21	0.3
	Poor	71	25	
T stage	0, 1	62	20	0.68
	2, 3, 4	98	26	
N stage	Negative	53	14	0.87
	Positive	107	32	
CRM	No	115	35	0.71
	Yes	45	11	
Resection Margins	No	133	37	0.84
	Yes	27	9	

ACC: adenocarcinoma, SCC: squamous cell carcinoma, CRM: circumferential resection margin.

**Table 5:** Postoperative complications and 30-day mortality.

	No (%) *n* = 117	<70 years	≥70 years	*P* value
Surgical Complications				
Wound infection	3 (2.6%)	3 (2.6%)		ns
Wound dehiscence	4 (3.4%)	3 (2.6%)	1 (0.9%)	ns
Chyle leak	6 (5.1%)	6 (5.1%)		ns
Anastomotic leak	10 (8.5%)	7 (6%)	3 (2.5%)	ns
Haemorrhage	3 (2.6%)	3 (2.6%)		ns
Resurgery	6 (5.1%)	6 (5.1%)		ns
Medical				
ARDS	4 (3.4%)	4 (3.4%)		ns
Atelectasis	14 (12%)	12 (10.2%)	2 (1.7%)	ns
LRTI	27 (23.1%)	18 (15.4%)	9 (7.7%)	ns
Pleural effusion	13 (11.1%)	1 (9.3%)	2 (1.7%)	ns
Arrythmias/A fib	23 (19.7%)	16 (13.7%)	7 (6%)	ns
Myocardial infection	2 (1.7%)	2 (1.7%)		ns
Postoperative deaths				
Yes	20	15	5	0.89
No	186	145	41	

ARDS: adult respiratory distress syndrome, LRTI: lower respiratory tract infection, A fib: atrial fibrillation.

**Table 6:** Univariate and multivariate analyses of demographic and histopathological characteristics.

	Univariate analysis			Multivariate analysis		
	*P* value	HR	95% CI	*P* value	HR	95% CI
Gender						
Age ≥70	0.00	2.27	1.52–3.38	0.00	2.12	1.42–3.14
T stage	0.29	1.017	0.69–1.49			
N stage	0.00	2.42	1.53–3.83	0.00	2.49	1.57–3.93
CRM	0.00	1.46	0.97–2.19			
Differentiation	0.43	0.95	0.66–1.37			
Positive resection margins	0.001	1.68	1.05–2.70	0.007	1.85	1.18–2.9
Recurrence	0.00	1.68	1.12–2.52	0.004	1.787	1.20–2.66

HR: hazard ration, CI: confidence interval, CRM: circumferential resection margins.
